# Management of Deep Neck Space Infections: A Large Tertiary Center Experience

**DOI:** 10.7759/cureus.34974

**Published:** 2023-02-14

**Authors:** Antonella Loperfido, Alessandro Stasolla, Cristina Giorgione, Fulvio Mammarella, Alessandra Celebrini, Gilberto Acquaviva, Gianluca Bellocchi

**Affiliations:** 1 Otolaryngology Unit, San Camillo Forlanini Hospital, Rome, ITA; 2 Neuroradiology Unit, San Camillo Forlanini Hospital, Rome, ITA

**Keywords:** infection, abscess, head and neck, dni, deep neck space infection

## Abstract

Introduction: Deep neck space infections (DNIs) represent serious bacterial infections affecting the deep cervical space and fascial planes of the neck. This study aims to describe our clinical and surgical experience in the management of DNIs, emphasizing the importance of appropriate imaging in the diagnostic setting and the role of the multidisciplinary approach according to the severity of the infection.

Methods: In this retrospective study, we describe 85 patients affected by DNIs coming to the Otolaryngology department observation from the Emergency Room of San Camillo Forlanini Hospital in Rome from January 2006 to December 2021 and treated both by pharmacological and surgical therapy.

Results: 54 patients (64%) were male, and 31 (36%) were female, with a mean age of 50.5 years. The most common cause of DNI was odontogenic, accounting for 70% of all collected cases. In 68 patients (80% of all cases), the surgical approach consisted of an extended unilateral cervicotomy, whereas in 17 patients (20% of all cases), a bilateral cervicotomy was performed. Surgical revision was required in 15 cases (18%). A tracheostomy was necessary in seven cases. The overall survival rate was 96.5%.

Conclusions: DNI represents a serious and life-threatening condition, remaining a constant challenge for the head and neck surgeon. Contrast-enhanced computed tomography is critical for therapeutic planning, which requires both a surgical approach and antibiotic therapy. Surgical treatment should be performed as soon as possible. In severe cases, the multidisciplinary approach is advisable.

## Introduction

Deep neck space infections (DNIs) are serious bacterial infections affecting the deep cervical space and fascial planes of the neck, often representing a diagnostic dilemma [1]. Some authors have noticed that DNIs frequency is currently lower than in the past for the availability of antibiotics [2].

Nevertheless, DNIs still represents an unsolved problem for physicians due to their severity, morbidity, and, especially when descending into the mediastinum, high mortality rates of 10%-40% related to sepsis and organ failure [3]. The main challenge is caused by the insidious evolution of this disease; in fact, a local infection, if inadequately treated, might potentially evolve into a DNI with the consequent possibility of descending mediastinitis or airway collapse. Abscesses, cellulitis, and phlegmons can spread along the fascial planes from the skull base to the mediastinum, causing severe and potentially life-threatening complications, including Descending Necrotizing Mediastinitis (DNM) [4].

The most common primary sources of DNIs are represented by odontogenic (35-42%) and pharyngotonsillitis infections. Other causes include sialoadenitis, foreign bodies, iatrogenic factors such as prior surgery and dental procedures, penetrating or blunt trauma to the head and neck district, jugular intravenous drug use, malignancies, lymphadenitis, and infected cysts [5].

The importance of a fast and accurate DNIs diagnostic and therapeutic strategy has been extensively addressed in the literature.

Regarding the diagnostic strategy, it is extremely important to make a differential diagnosis with other possible causes of neck swelling as metastatic lymph nodes [6]. With regard to the therapeutic approach, the principal points include managing the airways (often compromised), antibiotic therapy, DNI surgical incision, and drainage; finding and removing the possible cause of infection (such as periodontal diseases and dental caries or infected tonsils); and eventually treating of complications when present [7].

This study aims to describe our clinical and surgical experience in the management of DNIs, emphasizing the importance of appropriate imaging in the diagnostic setting and the role of the multidisciplinary approach according to the severity of the infection.

## Materials and methods

In this retrospective study, we describe 85 patients affected by DNIs coming to the Otolaryngology department observation from the Emergency Room of San Camillo Forlanini Hospital in Rome from January 2006 to December 2021 and treated both by pharmacological and surgical therapy. All procedures were performed in accordance with the ethical standards of our institutional ethical committee, and all patients signed informed consent.

Inclusion criteria were as follows: diagnosis of DNI based on the ENT physical examination with a fiberoptic laryngoscopy and the contrast-enhanced computed tomography (CECT); age>16 years; need for surgical drainage/debridement of the neck abscess in the operating room. When patients presented dyspnoea, stridor, hoarseness, dysphagia, and/or odynophagia or in case the fiberoptic laryngoscopy demonstrated a reduction of the respiratory space, the CT, which included head and neck district in all cases, was extended to the chest.

Patients with superinfected head and neck tumors, simple peritonsillar abscesses, superficial skin abscesses, and parotid abscesses were excluded from this retrospective study.

In all cases, the patients received antibiotic and steroid therapy. A broad-spectrum intravenous empirical antibiotic therapy such as penicillin, ceftriaxone, metronidazole, and/or clindamycin was administered to all patients, and it was later adjusted according to the microbiological findings. The steroid most administered was intravenous methylprednisolone.

All patients underwent surgery within 24 hours after accessing the emergency room. The surgical approach to DNIs included external transcervical incision and drainage of the abscess. Our surgical procedure includes horizontal neck incision of skin, subcutis, and platysma following a natural skin crease and sternocleidomastoid muscle isolation, providing access to potentially infected anatomical areas such as the submandibular, parapharyngeal, pterygoid, prelaryngeal, peritracheal, prevertebral, carotid, and lateral neck spaces. Subsequently, drainage of the identified purulent material and the removal of necrotic tissues were performed. The external drainage was maintained by placement of one or more drains as Penrose drains, glove finger, or Jackson-Pratt ones.

In cases of diffused DNI to the mediastinum, the surgical approach required the intervention of thoracic surgeons. Their intervention consisted in applying thoracic drains and/or performing thoracotomy in cases that involved more than one mediastinal compartment or extended beyond the upper mediastinum. In cases of dyspnea, the mediastinal spread of the infection, and/or prolonged postoperative intubation, a tracheotomy was performed.

After surgery, all patients were transferred to the intensive care unit for fluid and electrolyte resuscitation and close monitoring. A follow-up CT scan was performed 48 to 72 hours after surgery. In case of evidence of necrotic tissue, residual abscess, or unsatisfactory drainage, re-intervention was considered. Once circulatory and respiratory support was no longer needed, the patient was transferred from the intensive care unit to the otolaryngology department.

Data from the selected 85 cases were collated and processed using the Data Analysis ToolPak loaded in Excel (Microsoft®, Redmond, Washington, United States) to calculate descriptive statistics.

## Results

Of the 85 included patients, 54 (64%) were male, and 31 (36%) were female, showing a male prevalence with an M:F of 1.7:1. The mean age of patients was 50.5 years with a range from 16 to 85 years. The most common cause of DNI was odontogenic, accounting for 70% of all collected cases. The other identified sources were submandibular sialadenitis in 10 % of cases, peritonsillar abscesses in 3% of cases, prior neck surgery with a post-operative infection in 5% of cases, and a foreign body ingestion in 2% of cases, whereas the origin of the infection was not determined in 10% of all collected patients (Table 1).

**Table 1:** The primary origin of DNIs

Origin	Result
Odontogenic infections	70%
Sialadenitis	10%
Peritonsillar abscesses	3%
Postoperative infection	5%
Foreign body ingestion	2%
Unknown	10%

**Figure 1:**
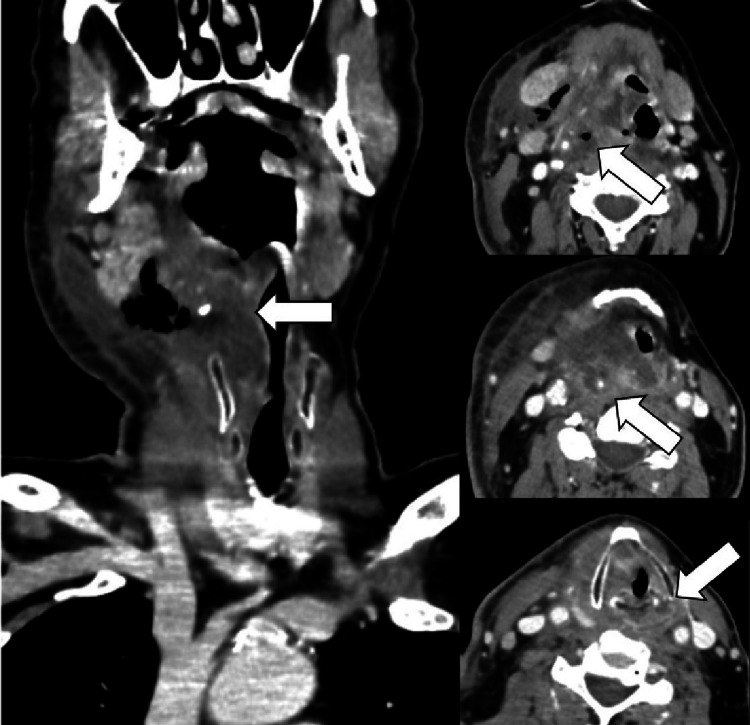
CECT imaging of a DNI on the right cervical side with interspersed gas collections involving submandibular, parapharyngeal, and retropharyngeal spaces with related aero-digestive tract displacement, superficial cellulitis signs, skin thickening, “dirty” appearance of subcutaneous fat and fluid collection into the platysma. The slight inflammatory enhancement of the right submandibular gland is well represented.

**Figure 2:**
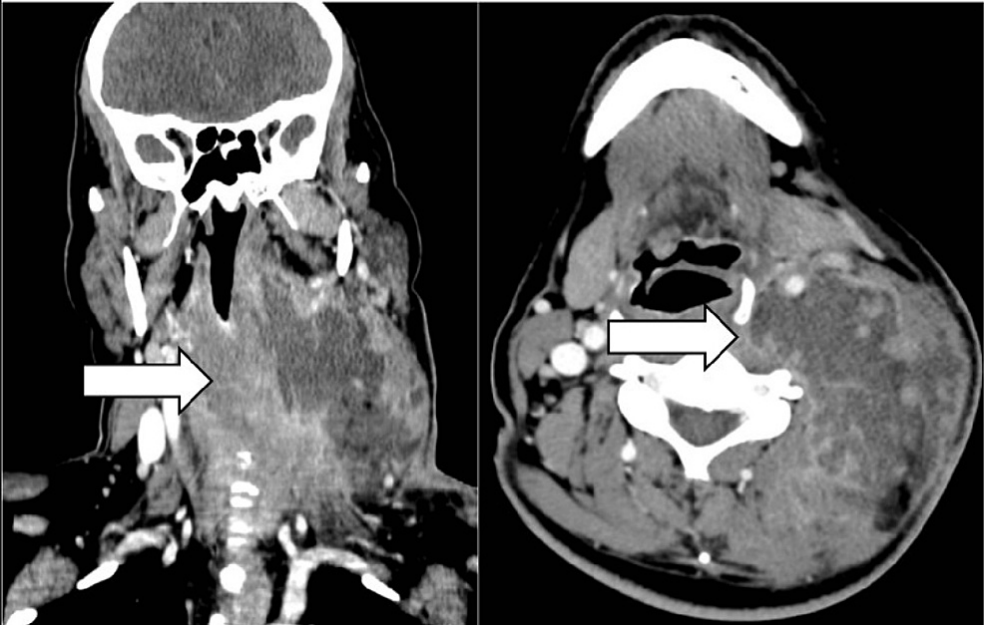
Large multiloculated abscess on the left cervical side involving the carotid space and the posterior triangle. CECT also identifies retropharyngeal edema, sternocleidomastoid myositis, internal jugular vein thrombosis, and slight compression of the proximal aero-digestive tract.

**Figure 3:**
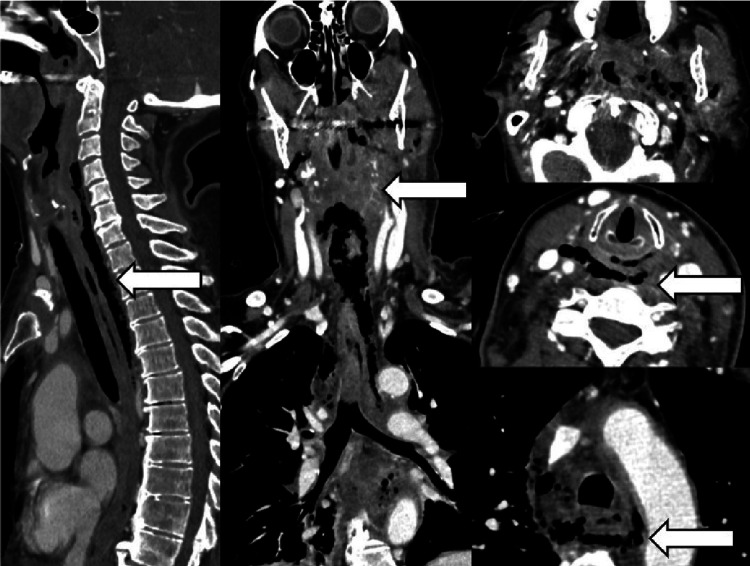
Left masticator and parapharyngeal space abscess complicating with descending necrotizing mediastinitis. CECT highlights the retrovisceral space dissection due to the presence of gas from the C3 to D5 level.

DNI was unilateral in 68 patients, and in 17 cases, the cervical infection presented multiple spaces of involvement until reaching the mediastinum. In Figure 1, we present a case of unilateral DNI; in Figure 2, a large multiloculated abscess; and in Figure 3, a case of abscess complicated by descending necrotizing mediastinitis.

In 68 patients (80% of all cases), the surgical approach consisted of an extended unilateral cervicotomy, whereas in 17 patients (20% of all cases), a bilateral cervicotomy was performed. In addition, the triggering cause was eliminated in those patients where the DNI origin was found and in the same contest of the surgical act. A microbiological swab on the purulent material was performed in all cases for the etiopathogenetic agent typing and the consequent antibiotic adjustment. In 10 cases (12%), the infection had spread to the prevertebral plane. In 14 cases (16%), drains were applied in the mediastinum due to the spread of the infection in the chest, with intervention by the thoracic surgeon. Moreover, extended mediastinal involvement required a thoracotomy by the thoracic surgeons in three patients. Surgical revision was required in 15 cases (18%), mainly due to the evidence of persistent signs of infection at the post-operative CT scan (14 patients). One patient was re-operated because of postoperative bleeding. The vascular-nerve bundle of the neck was preserved in all cases. A tracheostomy was necessary in seven cases. In all cases, the wound was healing with the first intention, except for one case in which a pectoralis major myocutaneous flap was required. In particular, the flap was necessary for a patient affected by DNI with mediastinal involvement treated by cervicotomy and thoracotomy and with consequent dehiscence at the lower sternum. In one case, there is a residual paresis of the perilabial muscles. The overall survival rate was 96.5%.

## Discussion

DNIs still represent a potentially life-threatening disease; therefore, an adequate diagnosis and prompt therapeutic management for these patients are essential. Descending necrotizing mediastinitis, septic shock, suppurative thrombophlebitis of the internal jugular vein associated with pulmonary septic embolism, thrombosis of the cavernous sinus, and carotid arterial erosion are the potentially lethal complications described, especially for immunocompromised patients or patients with comorbidities [8].

The results reported in our series are in line with data published on this topic by other authors. In fact, in our series, we found a male predominance of DNI patients, as widely recognized in many studies, even though the cause of such a prevalence remains largely unclear [9]. The mean age of our patients was 50.5 years, and this is consistent with the data presented in the literature [10].

In our paper, odontogenic infections represented the most common origin of DNI, accounting for 70% of all collected cases. This data is in line with most studies, which have confirmed that dental infection is the most common cause of neck abscesses [11]. These conditions are typically related to the lack of dental care due to the high costs of dentists, indifference, and poor knowledge about oral health with associated irregular tooth brushing [12].

Salivary gland infections represented the second most common infectious source in our collection. In the literature, several studies described the possible evolution of sialadenitis in DNI, especially for elderly and immunocompromised people [13]. In 10% of the cases that we collected, DNIs were related to infections of unknown origin, and this data is supported by recent literature reports showing that in up to 25% of cases of DNIs, it is not possible to identify the primary source of infection [14].

For a correct diagnostic approach, a complete head and neck physical examination with a flexible fiberoptic evaluation of the upper airway is required in all patients with potential DNI [15]. Leukocytosis is typically present in these patients due to the infection. A lack of leukocytic response may be induced by a concomitant viral infection, immune deficiency, drugs, or conditions such as cancer [16]. The role of secondary otalgia is another important point of great relevance to consider. In fact, atypical symptoms like reflective otalgia may mislead the clinical diagnosis [17].

Imaging plays a pivotal role in the evaluation of suspected DNI, and CT scans of the head and neck remain the gold standard for the evaluation of DNI because physical examination alone may misidentify the involved space and the number of involved spaces in 70% of cases [18]. When neck infection is hypothesized, CECT is the favorite investigation as it allows recognition, localization, and differential diagnosis between cellulitis/phlegmon and abscess and between suppurative lymph nodes and extranodal abscess collections; it can also identify mediastinitis and other complications and suggests the site of origin of the infection by detecting, e.g., dental infectious foci, ductal calculi of salivary glands, mandibular osteomyelitis, intratonsillar abscess [19]. Many inflammatory reactions include a phase of increased vascularity due to a loss of autoregulation, increased capillary permeability, and the formation of new capillaries. This can be demonstrated by the use of a contrast medium, which accumulates in inflamed tissues. Intravenous contrast also provides a clear visualization of bony and soft tissue structures of the head and neck, allows identification of the great neck vessels, and highlights the areas of inflammation, the collections, and the suspected complications [20]. The differential diagnosis between abscess and cellulitis/phlegmon is critical because surgical drainage is indicated only in the former case [21]. CECT recognizes an abscess as an encapsulated, hypodense, heterogeneous lesion with more hypodense central areas, with or without air bubbles. Specifically, the abscess may contain gas in case of communication with the superior aerodigestive pathway or due to the presence of gas-forming germs. Otherwise, cellulitis or phlegmon appears as a large, hypodense lesion with no peripheral wall and no purulent content or gas [22].

DNI requires a quick pharmacological treatment with intravenous antibiotics at the time of diagnosis because of the rapidly progressive nature of these infections. The therapy includes broad-spectrum antibiotics such as penicillin, ceftriaxone, metronidazole, and clindamycin, because of the mixed flora usually involved and to cover both aerobic and anaerobic bacteria [23]. Several authors also support steroid therapy in addition to antibiotics [24].

When antibiotics fail, abscess collection is more than 3 cm and extends to the deep neck spaces, and/or the patient presents complications such as airway impairment, sepsis, or descending infection, DNI surgical incision, and drainage is requested. The purpose of the surgical approach is to remove the triggering cause, drain the purulent collection, and save airway patency [25]. In the patients we described, surgery consisted of an extended unilateral or bilateral cervicotomy based on the degree of spread of the infection. An adequate surgical approach has a crucial role in the treatment of DNI, and in the literature, it is widely demonstrated that usually, intravenous antibiotics without surgery are insufficient. Cervical exploration and drainage, either by unilateral or bilateral cervicotomies, represents the currently accepted surgical treatment measure [26].

In 10 cases, the infection has spread to the prevertebral plane. This occurrence is uncommon, and it is due to the extension of the infection to the prevertebral space between the vertebrae bodies and prevertebral fascia [27]. In 17 patients, the DNI was complicated by the spread of the infection into the mediastinum, which required the intervention of a thoracic surgeon. Fourteen cases requested the application of drains in the mediastinum, while in three patients it was necessary to use a more invasive approach combining a cervicotomy with a thoracotomy to obtain a complete cervical, mediastinal, and pleural drainage. The importance of a multidisciplinary approach in the management of these patients among different specialties to get the most effective treatment for descending mediastinitis has been widely highlighted in the literature [28].

Fifteen patients required re-operation, mainly due to the evidence of persistent signs of infection at the postoperative CT scan. This concept underlines the importance of a CT scan in the follow-up, as it allows an early diagnosis and prompt therapy in case of persistence of the infectious process, with consequent patient prognosis improvement [29].

Seven patients required a tracheostomy to protect the airway due to the evidence of dyspnea, the mediastinal spread of the infection, and/or prolonged postoperative intubation. It has been widely stated that protecting the airway is critical in DNI management, and the most common therapeutical strategies to protect the airway are represented by orotracheal intubation and tracheostomy. In clinical practice, indications of tracheostomy include all difficult intubation conditions like the presence of signs of an obstructed upper airway, severe oral trismus, distorted anatomy of the neck, serious pharyngeal wall bulging, or critical laryngeal edema. Moreover, a common indication for tracheostomy is represented by prolonged intubation (more than two weeks) [30].

In our cohort wounds, mainly healed by first intention, and only in one case, a DNI with mediastinal involvement treated by cervicotomy and thoracotomy, a pectoralis major myocutaneous flap was required to solve residual dehiscence at the lower sternum. This technique has already been described in the literature [31].

In our collection, we reported 96.5% of overall survival. In particular, the spread of infection to the mediastinum is related to a poor prognosis. Prompt recognition and treatment of DNI are essential for an improved prognosis.

There are several limitations to our series as follows: first, the retrospective nature of this study; second, the number of patients, even though considerable if compared to other series published, is not sufficient to draw any conclusive evidence; finally, the long accrual period (15 years) with consequent lack of homogeneity in terms of clinical management among physicians involved.

## Conclusions

Deep neck space infections represent a serious and life-threatening condition, remaining a constant challenge for the head and neck surgeon. It requires a timely and correct diagnosis and treatment. Contrast-enhanced computed tomography is critical for therapeutic planning, which requires both a surgical approach and antibiotic therapy. Surgical treatment should be performed as soon as possible. In severe cases, the multidisciplinary approach is advisable.
